# Effects of Homogenization Heat Treatment on Microstructure of Inconel 718 Lattice Structures Manufactured by Selective Laser Melting

**DOI:** 10.3390/ma18174149

**Published:** 2025-09-04

**Authors:** Lucia-Antoneta Chicos, Camil Lancea, Sebastian-Marian Zaharia, Grzegorz Cempura, Adam Kruk, Mihai Alin Pop

**Affiliations:** 1 Department of Manufacturing Engineering, Transilvania University of Brasov, 500036 Brasov, Romania; l.chicos@unitbv.ro (L.-A.C.); zaharia_sebastian@unitbv.ro (S.-M.Z.); 2 Faculty of Metals Engineering and Industrial Computer Science, AGH University of Krakow, Al. Mickiewicza 30, PL-30 059 Krakow, Poland; cempura@agh.edu.pl (G.C.); kruczek@agh.edu.pl (A.K.); 3 Department of Materials Science, Transilvania University of Brasov, 500036 Brasov, Romania; mihai.pop@unitbv.ro

**Keywords:** Inconel 718, homogenization heat treatment, microstructure, selective laser melting, Laves phase, δ phase, γ’ and γ’’ phases, post-fabrication optimization

## Abstract

Inconel 718 is a nickel-based superalloy that has a wide range of applications in the industries that require corrosion resistance or high-temperature resistance. It is well known that parts display internal stresses, anisotropy, and alloying element segregation after the selective laser melting (SLM) process. A homogenization heat treatment, which reduces internal stresses and homogenizes the material structure, can resolve these shortcomings. The present study focuses on the impact of this heat treatment on the microstructure of the Inconel 718 material produced by SLM. The research results indicate that this heat treatment improves both the material microstructure and mechanical performance by lessening the microstructural inhomogeneities, dissolving the Laves phases, and promoting grain coarsening. The findings of this study can contribute to the optimization of post-fabrication strategies for Inconel 718 parts fabricated by SLM.

## 1. Introduction

Inconel 718 (IN718) is a nickel-based alloy that was developed by Huntington Alloys (which became part of Special Metals Corporation) for jet engines and gas turbines, because of its excellent high temperature properties and corrosion resistance [1]. With the advent of additive manufacturing technologies for metals, such as selective laser melting (SLM), the manufacture of parts with complex geometries made of IN718 has become increasingly popular. The use of SLM technology in manufacturing IN718 parts has led to the production of high-performance materials while also allowing for the production of parts with complex geometric configurations coupled with good mechanical properties [2,3,4]. IN718 is characterized by particularly good mechanical properties (high strength, good ductility, large fatigue resistance), high corrosion resistance, and good thermal resistance [5,6,7]. These special properties, coupled with moderate manufacturing costs, have led to the widespread use of this material in various industries where resistance to high temperatures and oxidation is crucial, such as the following: the aerospace industry (turbine elements, combustion chambers, and engine parts) [8,9,10], automotive (engine parts and exhaust systems) [11,12], the oil and gas industry (valves and piping systems that operate in highly corrosive environments) [13], and nuclear industries [14]. Despite its advantages, the selective laser melting (SLM) process has a notable drawback: the layer-by-layer deposition of molten material generates extremely high temperatures followed by rapid cooling, resulting in significant internal stresses and microstructural heterogeneities. This necessarily requires a stress-relieving and homogenizing heat treatment aimed at strengthening the mechanical characteristics of the material.

Compared to other metal alloys produced by SLM, such as alloys with high titanium concentrations, IN718 presents numerous advantages, among which the most important are its superior mechanical properties at high temperatures, lower cost, excellent weldability, and welded joints that have a high resistance to cracking. This last attribute regarding weldability makes it very suitable for additive manufacturing, which is quite comparable to the welding process [15]. Furthermore, its excellent corrosion resistance, even in particularly aggressive environments, increases the durability and longevity of the components compared to conventional stainless steels or titanium alloys in similar conditions of use. All these qualities make IN718 an extremely useful material for engineering applications that require resistance to corrosion or high temperatures, which also confers long-term reliability [13].

Despite the advantages offered by SLM, the rapid melting and solidification process can lead to a columnar grain structure aligned along the build direction, along with micro-segregation and Laves phase formation [16]. These non-equilibrium phases lead to anisotropic mechanical properties and can significantly degrade mechanical properties, reducing the material’s ductility and creep resistance, reducing overall performance.

Due to the specific SLM conditions, which include a series of high temperatures in the material deposition area, followed by abrupt cooling, the appearance of microstructural defects and internal stresses is unavoidable. For this reason, the parts produced by this method must undergo homogenization and stress relief heat treatment to improve the microstructure and material properties. This heat treatment serves to mitigate the difficulties outlined above by facilitating the diffusion of alloying elements and dissolving unwanted precipitates [17]. Thus, after the heat treatment, a more uniform microstructure, as well as better mechanical properties such as yield strength, tensile strength, and fatigue resistance, is obtained. By reducing microstructural flaws and eliminating unstable phases, homogenization heat treatment increases the long-term durability and mechanical performance of IN718 parts. Recent studies have shown that homogenization heat treatment contributes to grain recrystallization, lowers micro-segregation, and improves mechanical isotropy, resulting in good tensile strength, significantly increased ductility, and high thermal stability [18]. All of these enhancements will enable parts made of this material to function properly in a variety of key applications across industries.

This study extends prior research [19] and aims to demonstrate, through detailed analyses and both qualitative and quantitative evaluations, the impact of homogenization heat treatment on the microstructural evolution of the IN718 alloy. Accordingly, high-resolution characterization techniques were employed to investigate the microstructure and chemical composition of the as-fabricated and heat-treated samples, including scanning electron microscopy (SEM), Transmission and Scanning Transmission Electron Microscopy (TEM and STEM, respectively), and energy-dispersive X-ray spectroscopy (EDS).

## 2. Materials and Methods

### 2.1. SLM Fabrication of Samples

All samples feature a lattice structure with dimensions of 250 × 250 × 250 mm. The material of this study was gas-atomized IN718 alloy powder with spherical particles in a diameter range from 10 to 45 μm [5,20]. The manufacturing process of the samples was carried out at Nikon SLM Solutions AG in Lübeck, Germany.

**Figure 1:**
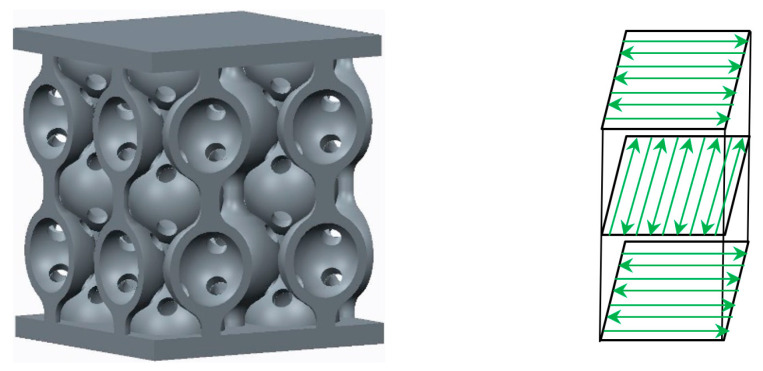
The geometry of the samples and the cross-snake manufacturing strategy [19].

The SLM process was carried out using an SLM 280HL system [21], equipped with two 400 W yttrium fibre lasers and a build volume measuring 280 × 280 × 350 mm^3^. The process parameters included a scanning speed of 900 mm/s, with a hatch distance of 120 μm and a layer thickness of 30 μm. For the directed energy deposition, the samples were vertically oriented, and a continuous cross-snake hatch strategy was used on the skin surface (Figure 1). The axis of deposition was parallel to the Z-direction according to ISO/ASTM52921-13 [22]. The manufacturing process was conducted in an argon inert gas environment, with a gas consumption rate of 2.5 L/min. The chemical composition of this metal powder is listed in Table 1.

### 2.2. The Heat Treatment of SAMPLES

**Figure 2:**
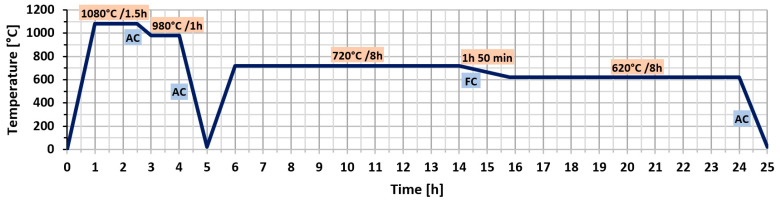
Schematic diagram of homogenization heat treatment process [23].

IN718 samples subjected to SLM were heat-treated in the Nabertherm™ L-245K2ANL Muffle Furnace with Flap Door (Nabertherm™, Nabertherm GmbH, Lilienthal (Germany)), max temperature: 1200 °C. The samples were heat-treated according to the following schedule, consistent with the industrial standard for casting IN718 [23,24]: homogenization treatment at 1080 °C for 1.5 h followed by air cooling (AC); solution treatment at 980 °C for 1 h with subsequent air cooling; and double ageing consisting of 8 h at 720 °C with furnace cooling (FC) at a rate of 55 °C/h down to 620 °C, followed by 8 h of air cooling. Figure 2 provides a graphical representation of the homogenization heat treatment schedule applied to the specimens.

Cutting; mounting in resin; grinding with SiC abrasive papers of 600, 1200, and 2000 grit sizes; and polishing in a diamond suspension on a LaboPol (Struers, Ballerup, Denmark) machine were all steps that were taken to get the samples ready for optical microscopic and SEM examinations. In order to reveal the microstructure, the specimens’ surfaces were etched using Kroll’s reagent (90 mL H_2_O, 6 mL HNO_3_, and 3 mL HF) [25].

A ZEISS Axio Imager optical microscope was used for optical microscopy (AIOM), and a scanning electron microscope (SEM, Merlin Gemini II of ZEISS, Oberkochen, Germany) equipped with an electron-dispersive spectrometer (EDS, Bruker Quantax 800, Ettlingen, Germany) was utilized for SEM analysis. The elemental composition was measured by energy-dispersive X-ray spectroscopy (EDS) [26]. The microstructure of the samples was characterized using a ChemiSTEM system-equipped scanning transmission electron microscope (STEM, Titan Cubed G2 60-300, FEI, Hillsboro, OR, USA) and a transmission electron microscope (TEM, Tecnai G2 20 TWIN, FEI) [26]. Energy-dispersive X-ray spectroscopy (EDS) mapping and STEM imaging with high-angle annular dark-field (HAADF) contrast were employed to investigate structural features down to the atomic scale. Phase identification was conducted using selected area electron diffraction (SAED) and further supported by EDS microanalysis, which was also utilized for elemental composition analysis.

Java Electron Microscopy Software (JEMS) (https://www.jems-swiss.ch/, accessed on 20 July 2025) was used to evaluate the electron diffraction pattern [27].

**Figure 3:**
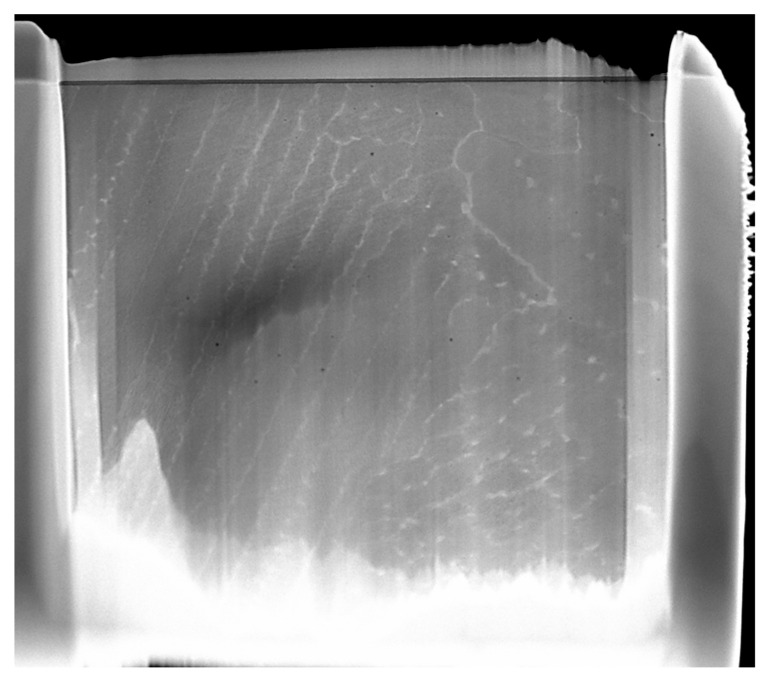
Lamella for TEM examination.

The NEON CrossBeam 40EsB (ZEISS) was utilized in the focused ion beam (FIB) technique to prepare the lamellae for TEM and STEM investigations. The procedure described in reference [28] was employed to prepare the region of interest (ROI) and to fabricate thin sections with a thickness ranging from 10 to 100 nm, as illustrated in Figure 3. The cross-section of the samples included all of the lamellae needed for TEM and STEM investigation (Figure 3).

## 3. Results and Discussion

**Figure 4:**
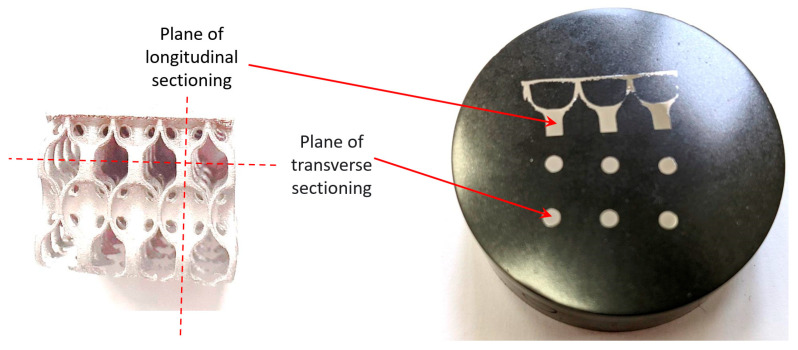
The sectioning planes of the IN718 samples subjected to SLM.

To analyze the microstructure of the samples, before and after the heat treatment, they were sectioned longitudinally and transversely (Figure 4) and then prepared for microscopic investigation, using the above-described procedures.

**Figure 5:**
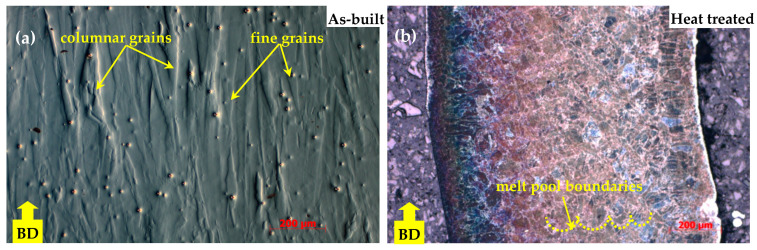
Microstructure of IN718 samples subjected to SLM in longitudinal section: as-built (**a**) and heat-treated (**b**).

**Figure 6:**
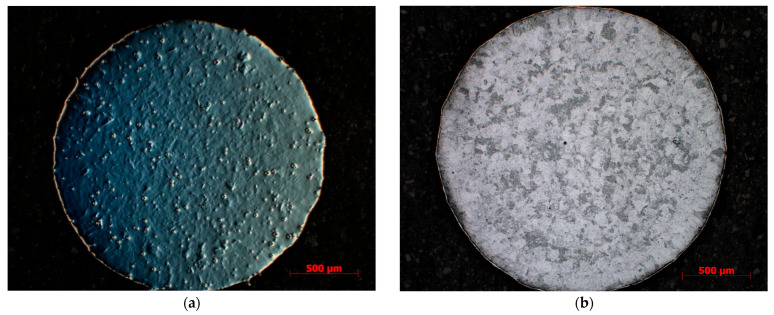
Microstructure of IN718 samples subjected to SLM in transversal section: as-built (**a**) and heat-treated (**b**).

**Figure 7:**
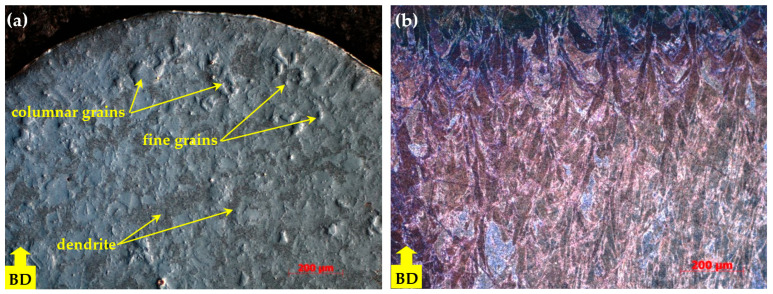
Microstructure of IN718 samples subjected to SLM in transversal section: as-built (**a**) and heat-treated (**b**) at higher magnification factor.

The microstructure of the as-fabricated IN718 subjected to SLM following examination with the ZEISS Axio Imager optical microscope is shown in Figure 5a, Figure 6a and Figure 7a, where a mixed-grain texture can be seen, which includes columnar grains, mainly oriented toward the building direction, and fine grains having varied orientation, due to the temperature gradient. In this regard, the columnar grains resulted from epitaxial growth in the centre of molten pools, while the fine grains resulted from the material’s heating history, due to the overlap of laser scanning at the edges of the molten pools [1]. Following the applied heat treatment, the grains tended to grow up, and the scanning stripes on plane XY disappeared after the heat treatment. The grain boundaries are clear now, and additional precipitations were observed in the grains and along the boundaries, as shown in Figure 5b and Figure 6b, and Figure 7b. At a magnification scale of 50×, the δ phase cannot be seen because of its small size.

**Figure 8:**
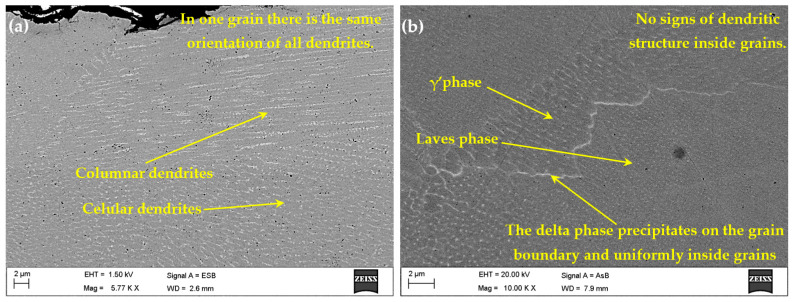
The microstructure of the IN718 as-built samples subjected to SLM in the longitudinal section at a lower (**a**) and higher (**b**) magnification factor.

Two sets of heat-treated and untreated samples were also examined through TEM analysis to analyze the microstructure in detail. A lamella for TEM analysis is shown in Figure 3 and was extracted from the cross-sections of both samples sets. When examining the microstructure of the as-built samples, the same orientation of all dendrites can be observed in Figure 8a. The particles with high Nb content, having a flower shape morphology, can be seen in the γ’ phase. The particles with a fine granulation morphology, with a high Fe content, make up the Laves phase, as shown in Figure 8b. The Laves phase and γ’ phase precipitates in the cellular and columnar dendrites are a result of the SLM manufacturing process.

**Figure 9:**
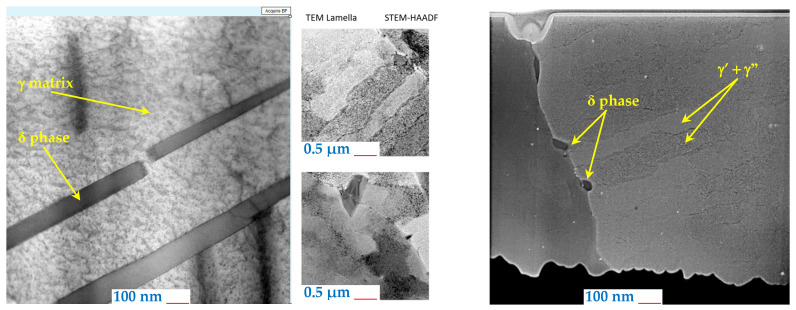
TEM microstructure analyses of the heat-treated IN718 samples subjected to SLM in the longitudinal section.

**Figure 10:**
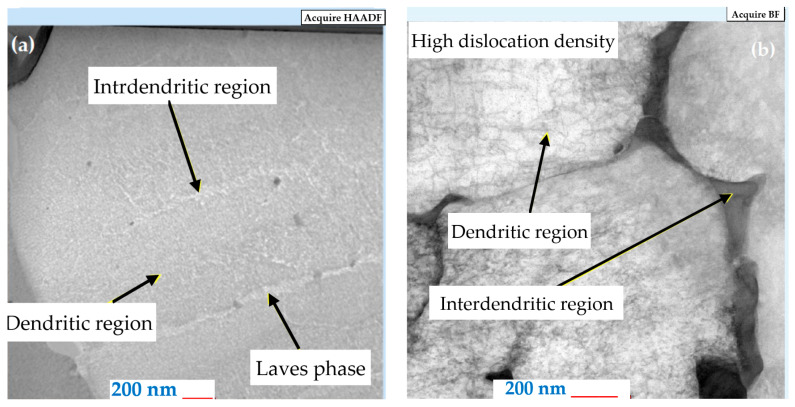
The microstructure of the IN718 as-built samples subjected to SLM in the longitudinal section at 200 nm magnification, Laves Phases (**a**) and dislocation density (**b**).

A higher magnification of the microstructure, given by the SEM images, also reveals the presence of elements that contribute to a decrease in the mechanical strength of the as-built material, as shown in Figure 9. In this regard, besides the Laves phase (Figure 10a), a significant dislocation density can also be observed (Figure 10b).

The Laves phase is harmful because it reduces the ductility of the material [28,29]. Therefore, the heat treatment is very important because it leads to the dissolution of this phase. The homogenization heat treatment process increases the percentage of the γ’ and γ’’ phases and also enhances the mechanical performance of the Inconel 718 superalloy because these phases are strengthening phases.

**Figure 11:**
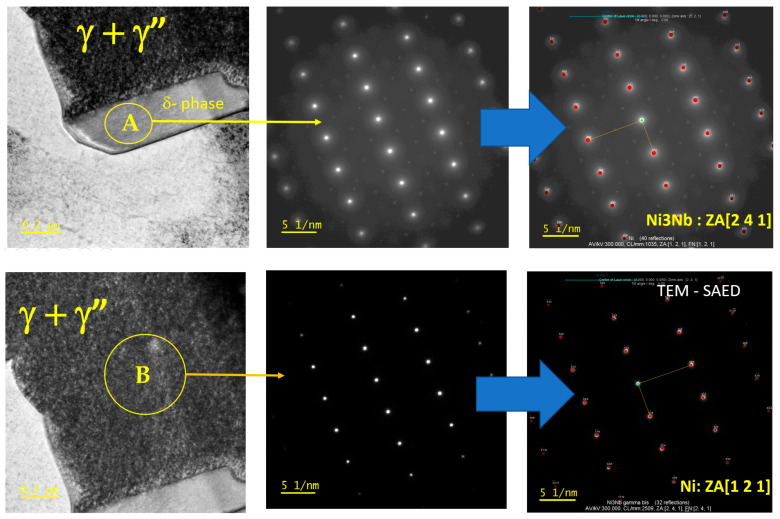
TEM- SAED diffraction pattern of the heat-treated IN718 samples subjected to SLM in the longitudinal section.

After the homogenization heat treatment process, the Laves and γ phases transform into γ’, γ’’, and δ phases. In this regard, γ’ and γ’’ phases were detected within the TEM analysis (Figure 9). In the same figure, the precipitation of the δ phase particles at the grain boundaries can also be observed. To confirm the nature and type of these precipitates [30], EDX analysis for the as-built and heat-treated samples was performed, and the results are shown in Figure 11.

The diffraction results (Figure 11) show that the light grey phase in region A is Ni3Nb, and the dark grey phase in region B is Ni, which verifies the conclusion of the above analysis. In addition to the above-presented aspects, it should also be mentioned that the structure obtained after the heat treatment is very similar to the Inconel 718 equilibrium structure [30].

**Figure 12:**
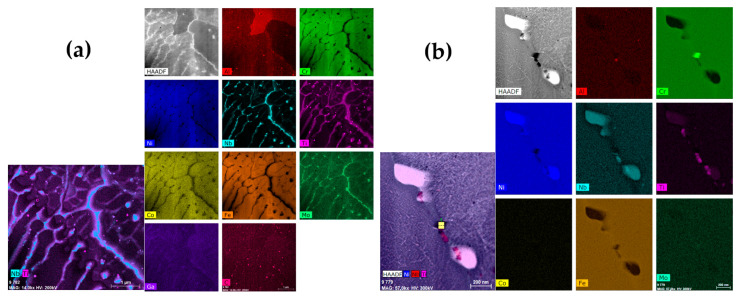
STEM-HAADF image of microstructure before (**a**) and after homogenization heat treatment (**b**) and corresponding EDS spectral images showing chemical element distribution maps.

**Figure 13:**
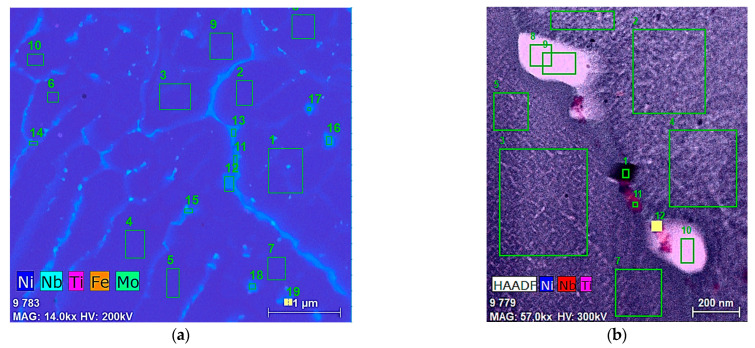
STEM-EDX elemental analysis image for major phases before (**a**) and after homogenization heat treatment (**b**).

In the as-deposited state (as illustrated in Figure 8), the solidification process leads to an enrichment in titanium (Ti), molybdenum (Mo), and niobium (Nb), leading to the formation of TiN, NbC, and the Laves phase. The presence of a Nb-rich intermetallic Laves phase harms the toughness and strength of the material [31]. After the heat treatment (as shown in Figure 7), elemental redistribution occurs, resulting in the development of δ phase needle-shaped formations, particularly in proximity to the partially dissolved Laves phase (Figure 12b and Figure 13). Additionally, the formation of the δ phase is associated with a reduction in chromium (Cr) and iron (Fe) concentrations within the dendritic interphase region [32].

Figure 13a shows the STEM-HAADF elemental analysis results for the major phases of the as-built material, and Figure 12b shows the elemental analysis results for the major phases after homogenization heat treatment.

Figure 13, Table 2 and Table 3 present the quantitative results of EDX analysis, before homogenization heat treatment, which shows a maximum ≈21% Cr and ≈21% Fe segregation at the selected spots (data collected at nineteen different spots). Table 3 shows the quantitative results of EDX analysis, after homogenization heat treatment, which shows a maximum ≈19% Cr and ≈17% Fe segregation at the selected spots (data collected at nineteen different spots).

After calculating the arithmetic averages of all mass percentages before and after heat treatment for Cr and Fe, we obtain 18.57 vs. 13.6% Cr and 16.36 vs. 12.33% Fe. An analysis of the mass percentages reveals a decrease in the concentrations of both iron and chromium in the heat-treated samples. This observation confirms the formation of the δ phase [33,34,35].

## 4. Conclusions

This study demonstrates that homogenization heat treatment significantly enhances both the mechanical properties and microstructure of the Inconel 718 material produced by selective laser melting. To investigate this, the microstructures and precipitates of the as-built and heat-treated Inconel 718 samples, fabricated via the SLM process, were analyzed using optical microscopy (OM), scanning electron microscopy (SEM), and transmission electron microscopy (TEM). The key findings and conclusions can be summarized as follows:

- After heat treatment, the Laves phase generates a δ phase in the vicinity due to the diffusion effect, and γ’ and γ’’ phases are also generated in the material structure. These phases contribute to the enhanced mechanical performance of the Inconel 718 superalloy due to their strengthening effect.
- Optical microscopy confirms the need for heat treatment by revealing a columnar dendritic structure caused by the rapid thermal cycles and radiative heat transfer in the SLM process.
- The quantitative results of EDX analysis allow for a simultaneous and quantitative original position statistical distribution analysis of different elements in the Inconel 718 alloy, before and after homogenization heat treatment. This analysis shows a maximum ≈21% Cr and ≈21% Fe segregation, at the selected spots. After homogenization heat treatment, the results of EDX analysis indicate a reduction in the mass percentages of these elements, with a maximum segregation of approximately 19% Cr and 17% Fe at the selected spots. These findings confirm the formation of the δ phase.
- A reduction in the mass percentages of these elements is further evidenced by the calculation of the arithmetic averages of all mass percentages before and after heat treatment. Specifically, the average mass percentages of Cr and Fe decrease from 18.57% to 13.6% and from 16.36% to 12.33%, respectively.
- Ageing heat treatment significantly enhances the microstructure and mechanical properties of additively manufactured IN718 by promoting grain growth, dissolving microstructural inhomogeneities, and reducing internal stresses.

The results presented in this study provide useful information for optimizing post-fabrication strategies applied to Inconel 718 material fabricated by SLM. By analyzing both the microstructural evolution and mechanical behaviour after heat treatment, the study provides useful information regarding the selection of thermal treatments that can improve the properties of the alloy. This is of particular importance because Inconel 718 is widely used in applications where mechanical strength, thermal stability, and structural homogeneity are of particular importance. Consequently, the results of this study can contribute to the development of more efficient and reliable manufacturing workflows in the field of additive manufacturing.

## Figures and Tables

**Table 1:** Chemical composition (in mass percent, wt%) of IN718 SLM ASTM F3055 powders used in this research [5].

	ASTM F3055/ASTM B637/AMS5664														
	Fe	Ni	Cr	Ta+Nb	Mo	Ti	Co	Al	Si	Mn	Cu	C	P	S	B
**Min.**	Bal.	50.00	17.00	4.75	2.80	0.65		0.20							
**Max.**		55.00	21.00	5.50	3.30	1.15	1.00	0.80	0.35	0.35	0.30	0.08	0.015	0.015	0.006

**Table 2:** STEM-EDX elemental analysis results for major phases before homogenization heat treatment—mass percentages.

Zone No.	Al-K	Cr-KA	Ni-KA	Nb-KA	Ti-KA	Co-KA	Fe-KA	Mo-KA
1	1.35	22.14	50.08	3.72	1.23	0.38	18.38	2.72
2	1.35	20.18	52.86	2.84	1.02	0.37	18.84	2.55
3	2.47	20.01	51.83	3.28	1.14	0.37	18.36	2.55
4	1.55	20.18	52.77	2.72	0.9	0.37	18.98	2.54
5	1.54	20.48	51.96	2.59	0.81	0.37	19.72	2.52
6	1.33	21.24	50.62	2.15	0.7	0.35	21.19	2.42
7	1.5	20.4	52.28	2.92	0.97	0.37	18.94	2.62
8	1.96	20.15	52.22	2.95	1.07	0.38	18.72	2.56
9	2.55	20.06	51.67	3.32	1.18	0.37	18.25	2.59
10	1.37	20.53	52.88	2.3	0.75	0.36	19.45	2.36
11	1.2	16.13	46.82	16.33	2.18	0.34	12.74	4.26
12	1.42	15.96	47.24	15.59	2.32	0.35	12.97	4.16
13	1.27	15.98	46.02	17.03	2.35	0.35	12.76	4.23
14	1.4	18.88	51.1	7.21	1.74	0.3	16.27	3.09
15	1.05	15.87	45.05	18.38	2.11	0.32	12.75	4.47
16	1.08	15.53	45.94	18.13	2.12	0.37	12.7	4.03
17	1.34	15.36	46.39	17.86	2.17	0.37	12.52	4
18	1.22	15.96	45.36	17.49	2.61	0.32	12.8	4.23
19	1.4	17.83	49.46	10.68	1.98	0.36	14.56	3.73

**Table 3:** STEM-EDX elemental analysis results for major phases after homogenization heat treatment—mass percentages.

Zone No.	Al-K	Cr-KA	Ni-KA	Nb-KA	Ti-KA	Co-KA	Fe-KA	Mo-KA
1	5.93	19.28	50.48	12.44	0.81	0.51	5.71	4.84
2	3.92	19.18	48.74	4.27	1.63	0.99	17.5	3.78
3	3.99	19.04	48.69	4.26	1.71	1.02	17.45	3.84
4	4.01	19.14	48.81	4.23	1.6	0.99	17.45	3.78
5	3.92	19.1	48.81	4.21	1.64	1.02	17.52	3.79
6	4.08	19.33	48.79	4.03	1.57	0.99	17.55	3.66
7	4.1	6.19	52.36	14.17	1.72	1.03	17.62	3.82
8	3.57	2.24	54.09	20.39	3.45	0.96	12.28	3.03
9	4.62	10.6	53.74	19.91	3.46	0.96	3.67	3.03
10	4.95	9.97	53.41	20.81	3.63	0.88	3.34	3.02
11	4.55	9.98	51.25	20.21	14,01	0.78	9.22	4.01
12	4.14	9.14	42.32	17.44	13.31	0.88	8.65	4.12

## Data Availability

The original contributions presented in this study are included in the article. Further inquiries can be directed to the corresponding author.
